# Effects of personality on overtime work: a cross-sectional pilot study among Japanese white-collar workers

**DOI:** 10.1186/1756-0500-7-180

**Published:** 2014-03-27

**Authors:** Mitsuo Uchida, Minoru Kaneko, Shigeyuki Kawa

**Affiliations:** 1 Center for Health, Safety and Environmental Management, Shinshu University, 3-1-1 Asahi, Matsumoto 390-8621, Japan

**Keywords:** Big Five, Long work hours, Overtime work, Personality, White-collar workers

## Abstract

**Background:**

As detailed associations between personality and long work hours are unclear, we assessed associations between personality dimensions and overtime work among Japanese white-collar workers.

**Methods:**

From records of hours worked over 12 months by 267 office workers in an organization within the service industry, average overtime work hours per month and occurrence of excessive overtime was determined for each worker. Excessive overtime was defined as >  45 overtime work hours per month for at least one month. Responses to a questionnaire assessing socio-demographic and workplace-related factors and the Big Five personality test were analyzed. Associations between personality factors and overtime work were assessed by multivariate logistic regression analysis.

**Results:**

Low Extraversion was associated with excessive overtime work (OR 2.02, 95%CI 1.02 – 4.02, *P* =  0.04).

**Conclusions:**

It is suggested that workers with low Extraversion can’t share work when busy to avoid excessive overtime. Personality factors should be considered in studies evaluating work time. Moreover, strengthening communication among workers with low Extraversion may reduce excessive overtime work and associated health problems.

## Background

Number of work hours is important in occupational health [1-3]. Long work hours are associated with adverse health effects, including cardiovascular disease [4-6], high blood pressure [7], mortality [8], and work-related injuries [9]. These adverse effects are mainly caused by sleep loss and severe fatigue due to extended work hours [10,11]. Therefore, excessive work hours should be discouraged or limited to prevent adverse health effects.

As long work hours are thought to affect work-related diseases, official guidelines have been developed in Japan with the aim of preventing health problems due to long work hours [12]. Every year, the Ministry of Health Labour and Welfare releases data detailing industrial accidents [13] wherein we can see a relationship between long work hours and ill-health. These data show excessive work hours consistently associated with physical diseases such as cerebrovascular or cardiovascular diseases, but not with mental disorders. Several studies have been conducted to explore associations between long work hours and mental health, however, reviews show results were inconsistent and associations unclear [14,15]. This disparity may be because workers’ mental health is affected by not only number of working hours but also their relationships at work [16], family factors [17] and individual factors [15]. The number of working hours, therefore, may be only one factor among several affecting workers’ mental health and it is necessary to clarify the associations in detail.

In general, we see variation in number of working hours by occupation or workplace [18,19]. However, we might see this variation among workers at the same workplace doing the same job. Though this phenomenon occurs, few reports address the reasons based on scientific theory. Although some reports have shown work hour variation is related to workload [20,21], position [22], immersion [23,24] or type A personality [6,25-27], there is little information relating number of working hours to occupational health. This paucity of information may be one reason why the association between number of work hours and mental health is unclear.

Personality is known to be an important factor in human behavior and mental health. Recently a significant body of literature has accumulated to support the five-factor theory of personality in which factors such as Extraversion or Conscientiousness have been shown to be associated with symptoms of depression [28-30], work performance [31,32] or absenteeism of workers [33]. Therefore, personality factor is speculated to be also associated with number of working hours. However, as far as the authors are aware, there are no reports clarifying the association between Big Five personality and number of working hours. If Big Five personality factors are associated with number of working hours, particular personality characteristics may play important roles in the association between number of working hours and workers’ mental health. Thus, personality of workers is thought to be fundamental information to understanding the association.

Before planning a study regarding number of working hours and workers mental health, we thought to clarify the association between personality and number of working hours. Therefore, the aim of this study is to examine which Big Five factors are related to long work hours. Moreover, findings of this study may inform construction of further measurements of long work hours. Thus, we conducted this pilot study to examine associations between workers’ scores on the Big Five personality test and number of working hours.

## Methods

### Subjects

A total of 467 office workers employed in a service industry organization in August 2012 participated in this study. They worked during the day almost always seated at a personal computer, performing similar tasks such as making documents, responding to telephone calls, filing materials, and attending meetings. At the beginning of the study, leaflets explaining the study procedure were distributed to all workers. Written informed consent was obtained from all participants. All data were provided and analyzed anonymously. Of the 467 workers, 330 (70.7%) completed the questionnaire. After excluding 31 workers for whom complete work hour information was unavailable, 18 higher level managers who did not record their work time, 8 workers with severe illnesses, and 6 workers who submitted incomplete questionnaires, 267 workers were enrolled (57.2%). The study design and procedure were reviewed and approved by the Committee for Medical Ethics of Shinshu University (approval number 1790).

### Questionnaires

A self-administered questionnaire comprising items regarding socio-demographic factors, workplace-related factors, and Big Five personality factors was distributed to every participant. Participants completed and immediately returned the questionnaire in a sealed envelope. The questions were based on those in previous published reports [20,24,34,35]. Socio-demographic factors included sex (male, female), age (years), height (cm), weight (kg), average sleep per night (hours), drinking alcoholic beverages (every day, sometimes, occasionally, never), smoking (current smoker, ex-smoker, never smoker), physical exercise (every day, sometimes, occasionally, never), hobbies (yes, no), meal times (regular, irregular), current illness (yes, no), marital status (married, unmarried (including divorced or widowed)), educational level (postgraduate, graduate, graduate of junior college or technical training, no academic qualifications), stress at home (yes, no), and human relations at home (good, poor). Workplace-related factors included position within the company (manager or higher, assistant manager, chief, clerk, part-time worker) and commuting time (minutes). The factors also included job demand, decision latitude, and social support [36,37] according to the Japanese version of Brief Job Stress Questionnaire [38]. In addition, work-time related factors were assessed using the following negative and positive attitude questions. Negative factors: can you take paid holidays?; do you have an opportunity to stop work to go home?; do you have difficulty going home due to concern about other workers?; do you have a fear of the boss?: Positive factors: do you have work motivation?; do you have a work purpose?; do you have success motivation?; do you feel gratitude for your employment? These questions were also based on those in previous reports [20,24,34]. These factors were assessed on a 4-point Likert scale (ranging from a score of 4 for high and 1 for low). The internal consistencies (Cronbach’s alpha) of the Negative and Positive attitude questions were 0.55 and 0.57, respectively.

We assessed personality characteristics using the Big Five personality test [39], based on the five-factor model which is generally accepted worldwide. The model describes an individual’s personality along five dimensions, Extraversion, Agreeableness, Conscientiousness, Emotional stability, and Openness. In this study, the Japanese version of the Big Five personality test [39] including 70 questions was applied. Scores for each characteristic range from 0 to 12 with 12 indicating a high degree of the characteristic. Scores were determined using Windows software accompanying the manual [39] and applied for analysis in this study. In this study, the internal consistencies (Cronbach’s alpha) of Extraversion, Agreeableness, Conscientiousness, Emotional stability and Openness were found to be 0.90, 0.76, 0.84, 0.91 and 0.81, respectively.

### Number of working hours

In this sample, full-time workers were contracted to work from 08:30 to 17:15 daily with a 1 h lunch break amounting to a standard 7 h 45 min/day, 38 h 45 min/week and part-time workers were contracted to work a standard 30 h/week. The principal of the organization encouraged all workers to work a limited number of overtime hours. Additional work hours beyond the standard were counted as overtime work hours. All workers recorded their working time into their personal computers daily to be checked by their boss, thus allowing accurate determination of the number of hours worked by each subject. Such data recorded for the previous 12 months were obtained and used in this study.

As adverse health effects can be caused by not only permanent long work hours but also accumulation of overtime hours within a short period [12], two outcome measures were employed to evaluate number of working hours. First, average number of overtime work hours, calculated as the average of 12 months of overtime for each worker and expressed as “hours per month”, and second, excessive overtime work. In Japan, workers can work overtime by either working beyond the set number of hours per day or by working on their scheduled day off, to a maximum of 45 h/month, as prescribed by labor-management agreements; if these agreements include special clauses, workers can work more than this upper limit [40]. Consequently, all workers are aware of their upper limit for overtime work hours, therefore, it is important to know whether workers breach the limit or not when evaluating long work hours. In this study, excessive overtime work was defined as that exceeding 45 h/month at least once during the previous 12 months.

### Statistical analysis

Participants were divided into 4 groups by age (20 – 29, 30 – 39, 40 – 49, ≥ 50 years), 3 by BMI (< 18.5, 18.5 – 25, ≥ 25), and 4 by work status (Assistant manager or higher, Chief, Clerk, Part-time worker). Other factors were dichotomized (yes/no, Likert scale 1 – 2/3 – 4 or suitable division according to preliminary calculations). In this study, each personality factor was dichotomized by median score into high-score group or low-score group to determine whether high personality score group did more overtime work than low personality score group or otherwise, respectively. Average numbers of overtime work hours were similarly treated with participants classified as long overtime work group or short overtime work group according to median overtime work hours. Subjects were classified as excessive overtime work occurrence group if they worked overtime beyond 45 h/month at least once during 12 months or excessive overtime work non-occurrence group if they did not. For statistical analysis, univariate and multivariate logistic regression models were used. First, a univariate regression model of socio-demographic and workplace-related factors for the two outcome measures were used as preliminary analysis. Subsequently, significant factors which were observed in the first step were used as adjusting factors for a multivariate regression model, and associations between the Big Five personality factors and the two outcome measures were evaluated. All dimensions of personality factors were analyzed independently to avoid overadjustment [33]. Associations were expressed as odds ratios (OR) and 95% confidence interval (95%CI). PASW 18.0 software (SPSS Inc., Chicago, IL) was used for all analyses, and *P* <  0.05 was taken to indicate statistical significance.

## Results

Table 1 shows details of the participants who comprised 142 male and 125 female workers, with most (38.2%) aged 30 – 39 years old. Participants worked a mean  ±  SD of 17.8  ±  13.0 overtime work hours per month over 12 months, with a median of 16 h (range 1.0 – 66.1 h). Workers were dichotomized into long overtime work group and short overtime work group 50% each according to the median (16 h) overtime work hours. Seventy-four participants (27.7%) worked excessive overtime at least once during the previous 12 months. Table 2 shows the descriptive of the Big Five personality factors of participants. The scores were not distributed normally and the median scores were used to divide responses into two groups.

**Table 1:** Socio-demographic and workplace-related factors of the study population

**Factor**	**Classification**	**n = 267**	**%**
Socio-demographic factors			
Sex	Male	142	53.2
	Female	125	46.8
Age	20-29	35	13.1
	30-39	102	38.2
	40-49	76	28.5
	50-	54	20.2
BMI	<18.5	32	12.0
	18.5-25	186	69.7
	≥25	49	18.4
Sleep	<6 hours	37	13.9
	≥6 hours	230	86.1
Drinking	Never or occasionally	170	63.7
	Sometimes or more often	97	36.3
Smoking	Never or ex-smoker	226	84.6
	Current smoker	41	15.4
Physical exercise	Never or occasionally	121	45.3
	Sometimes or more often	146	54.7
Hobbies	Yes	184	68.9
	No	83	31.1
Meal times	Regular	232	86.9
	Irregular	35	13.1
Current illness	Yes	67	25.1
	No	200	74.9
Marital status	Married	174	65.2
	Unmarried	93	34.8
Educational level	Graduate or higher	139	52.1
	Under graduate	128	47.9
Stress at home	Yes	103	38.6
	No	164	61.4
Human relations at home	Good	242	90.6
	Poor	25	9.4
Workplace-related factors			
Position	Assistant manager or higher	23	8.6
	Chief	69	25.8
	Clerk	94	35.2
	Part-time worker	81	30.3
Commuting time	<30 minutes	178	66.7
	≥30 minutes	89	33.3
Job demand	High	179	67.0
	Low	88	33.0
Decision latitude	High	231	86.5
	Low	36	13.5
Social support	High	196	73.4
	Low	71	26.6
Taking paid holiday	Yes	218	81.6
	No	49	18.4
Stopping work	Yes	102	38.2
	No	165	61.8
Difficulty going home	Yes	55	20.6
	No	212	79.4
Fear of the boss	Yes	59	22.1
	No	208	77.9
Work motivation	Yes	226	84.6
	No	41	15.4
Work purpose	Yes	213	79.8
	No	54	20.2
Success motivation	Yes	54	20.2
	No	213	79.8
Gratitude for employment	Yes	226	84.6
	No	41	15.4

**Table 2:** Descriptive of the Big Five personality factors for the study population

**Big Five personality factors**		
Extraversion	4.4	± 4.0
	3	(0–12)
Agreeableness	8.7	± 2.6
	9	(0–12)
Conscientiousness	6.5	± 3.5
	7	(0–12)
Emotional stability	5.7	± 4.2
	5	(0–12)
Openness	3.7	± 3.1
	3	(0–12)

Personality factor scores and overtime work hours are shown as mean ± SD and median (range).

Table 3 shows the univariate logistic regression model with, 8 factors, sex, BMI, meal times, position, job demand, taking paid holiday, stopping work, and fear of the boss that were significantly associated with both of the two outcome measures. In addition, 4 factors, age, marital status, decision latitude and difficulty going home were significantly associated with one of the two outcome measures. Four personality factors, Extraversion, Agreeableness, Conscientiousness and Emotional stability were significantly associated with excessive overtime work in this univariate model.

**Table 3:** Results of univariate logistic regression analysis for long overtime work hours and excessive overtime work

**Factor**	**Classification**	**Long overtime work hours**				**Excessive overtime work**			
		**OR**	**95% CI**		***P***	**OR**	**95% CI**		***P***
Socio-demographic factors									
Sex	Male	1.00				1.00			
	Female	0.18	0.10	0.30	<0.01	0.26	0.14	0.47	<0.01
Age	20-29	1.00				1.00			
	30-39	0.67	0.31	1.45	0.31	0.69	0.31	1.52	0.35
	40-49	0.57	0.25	1.28	0.17	0.31	0.13	0.76	0.01
	50-	0.69	0.29	1.64	0.40	0.58	0.23	1.42	0.23
BMI	18.5-25	1.00				1.00			
	<18.5	0.65	0.30	1.40	0.27	0.66	0.26	1.71	0.40
	≥25	2.22	1.15	4.32	0.02	1.98	1.03	3.83	0.04
Sleep	≥6 hours	1.00				1.00			
	<6 hours	0.92	0.46	1.85	0.82	1.72	0.83	3.57	0.14
Drinking	Never or occasionally	1.00				1.00			
	Sometimes or more often	0.95	0.57	1.56	0.83	0.86	0.49	1.51	0.59
Smoking	Never or ex-smoker	1.00				1.00			
	Current smoker	1.48	0.75	2.90	0.26	1.09	0.53	2.28	0.81
Physical exercise	Sometimes or more often	1.00				1.00			
	Never or occasionally	0.84	0.52	1.36	0.47	1.12	0.65	1.91	0.69
Hobbies	Yes	1.00				1.00			
	No	1.01	0.60	1.70	0.96	0.92	0.51	1.64	0.77
Meal times	Regular	1.00				1.00			
	Irregular	3.91	1.71	8.97	<0.01	3.82	1.84	7.94	<0.01
Current illness	No	1.00				1.00			
	Yes	0.68	0.39	1.19	0.18	0.55	0.28	1.08	0.08
Marital status	Married	1.00				1.00			
	Unmarried	1.32	0.79	2.18	0.29	1.78	1.03	3.09	0.04
Educational level	Graduate or higher	1.00				1.00			
	Undergraduate	0.74	0.46	1.20	0.22	0.66	0.38	1.14	0.14
Stress at home	No	1.00				1.00			
	Yes	0.65	0.39	1.06	0.08	0.88	0.51	1.54	0.66
Human relations at home	Good	1.00				1.00			
	Poor	0.90	0.39	2.05	0.80	1.25	0.52	3.05	0.62
Workplace-related factors									
Position	Clerk	1.00				1.00			
	Chief	0.92	0.35	2.41	0.87	0.83	0.33	2.11	0.70
	Assistant manager or higher	1.12	0.58	2.20	0.73	0.61	0.32	1.16	0.13
	Part-time worker	0.05	0.02	0.13	<0.01	0.03	0.01	0.14	<0.01
Commuting time	<30 minutes	1.00				1.00			
	≥30 minutes	0.80	0.48	1.33	0.39	0.80	0.45	1.42	0.44
Job demand	Low	1.00				1.00			
	High	3.15	1.84	5.41	<0.01	2.67	1.39	5.11	<0.01
Decision latitude	High	1.00				1.00			
	Low	1.90	0.92	3.93	0.08	2.08	1.01	4.30	0.048
Social support	High	1.00				1.00			
	Low	1.28	0.74	2.21	0.37	1.63	0.91	2.93	0.10
Taking paid holiday	Yes	1.00				1.00			
	No	5.01	2.38	10.55	<0.01	4.47	2.33	8.54	<0.01
Stopping work	No	1.00				1.00			
	Yes	2.42	1.45	4.02	<0.01	1.97	1.14	3.40	0.01
Difficulty going home	No	1.00				1.00			
	Yes	3.32	1.73	6.37	<0.01	1.85	0.99	3.47	0.05
Fear of the boss	No	1.00				1.00			
	Yes	2.09	1.15	3.81	0.02	2.63	1.43	4.82	<0.01
Work motivation	Yes	1.00				1.00			
	No	1.31	0.67	2.56	0.43	1.26	0.61	2.58	0.54
Work purpose	Yes	1.00				1.00			
	No	0.98	0.54	1.78	0.95	1.40	0.74	2.67	0.30
Success motivation	Yes	1.00				1.00			
	No	0.58	0.31	1.07	0.08	0.89	0.46	1.72	0.73
Gratitude for employment	Yes	1.00				1.00			
	No	0.93	0.48	1.80	0.82	0.59	0.26	1.34	0.21
Big Five personality factors									
Extraversion	High	1.00				1.00			
	Low	1.57	0.97	2.55	0.07	3.07	1.73	5.45	<0.01
Agreeableness	High	1.00				1.00			
	Low	1.35	0.83	2.19	0.22	2.06	1.18	3.61	0.01
Conscientiousness	High	1.00				1.00			
	Low	1.27	0.78	2.06	0.33	1.75	1.01	3.05	0.047
Emotional stability	High	1.00				1.00			
	Low	1.48	0.91	2.40	0.11	2.49	1.42	4.37	<0.01
Openness	High	1.00				1.00			
	Low	1.23	0.76	2.00	0.39	1.36	0.79	2.35	0.26

Table 4 shows associations between personality and long overtime work hours and excessive overtime work. Twelve factors found significant in the univariate model plus the social support factor which is usually employed in occupational health surveys, comprised the 13 factors used as adjusting factors. We made correlation matrices within the Big Five personality factors and these 13 factors and no multicollinearity was found among them (the largest correlation was found between Emotional stability and fear of the boss; r = −0.24). Extraversion was found to be significantly associated with excessive overtime work (OR 2.02, 95%CI 1.02 – 4.02, *P* =  0.04), whereas the other four Big Five personality factors showed no associations in either model.

**Table 4:** Odds ratios (OR), 95% confidence intervals (95%CI) and significance of personality factors for belonging to the group with long overtime work hours and excessive overtime work

**Personality factor**		**Long overtime work hours**				**Excessive overtime work**			
		**OR**	**95% CI**		***P***	**OR**	**95% CI**		***P***
Extraversion	High	1.00				1.00			
	Low	0.88	0.46	1.68	0.70	2.02	1.02	4.02	0.04
Agreeableness	High	1.00				1.00			
	Low	0.83	0.43	1.58	0.57	1.69	0.85	3.36	0.13
Conscientiousness	High	1.00				1.00			
	Low	0.94	0.48	1.84	0.85	1.51	0.75	3.06	0.25
Emotional stability	High	1.00				1.00			
	Low	0.66	0.34	1.28	0.22	1.54	0.77	3.06	0.22
Openness	High	1.00				1.00			
	Low	0.75	0.38	1.50	0.42	1.14	0.56	2.33	0.72

Each personality factor was adjusted for any socio-demographic factor or work-place related factor significant in univariate model. Adjusted factors were sex, age, BMI, meal times, marital status, position, job demand, decision latitude, social support, taking paid holidays, stopping work, difficulty going home and fear of the boss.

## Discussion

In this pilot study, associations between the Big Five personality factors and long overtime work hours and excessive overtime were evaluated among Japanese white-collar workers. Low Extraversion was found to be associated with excessive overtime work, while it had no significant association with long overtime work hours.

We employed the globally accepted Big Five personality test [39] to evaluate the characteristics of workers in this study. Although previous studies showed associations between Extraversion and work performance of workers [32] or absenteeism [33], there have been no previous studies regarding the association with number of working hours. The results of the present study indicated that low Extraversion was related to excessive overtime in workers in both univariate and multivariate regression models. The association was significant for excessive overtime work but not daily longer overtime work hours. Extraversion generally indicates a personality that displays behaviors of talkativeness, liveliness or proactivity [39]. We therefore speculate that the present findings could be explained to the extent that workers with the characteristic of low Extraversion may tend to work alone even when they become busy, which may result in excessive overtime work as their work burden increases. In addition to this result, a previous review [28] and other studies [29,30] showed an association between lower Extraversion and depressive symptoms. From these results, low Extraversion may be associated with both excessive overtime and symptoms of depression. The present study may therefore be useful to inform the relationship among number of working hours and mental health. As Conscientiousness was also shown to be related to high work performance [31], we initially expected that this characteristic would be associated with overtime work, but no such association was found in the multivariate analysis. The absence of this association could be explained that conscientious workers may tend to rigidly follow the rules regarding the number of permissible overtime working hours. An individual with the Conscientiousness personality trait not only performs his/her work conscientiously but also behaves honestly. Agreeableness, Emotional stability and Openness were found to be associated with depressive symptoms in other studies [29,30]. In this study, Agreeableness and Emotional stability were associated with excessive overtime work in the univariate analysis, but this association was not evident in the multivariate analysis. This phenomenon indicated that low association may exist between Agreeableness and Emotional stability and excessive overtime work in univariate analysis. But those associations disappeared by adjustment in multivariate analysis because some confounding variables might exist between Agreeableness and Emotional stability and excessive overtime work. In other words, perhaps these particular personality factors may associate with mental health directly rather than excessive overtime work. However, these possibilities are not explained by this study alone, further study is needed to clarify associations between number of working hours and mental health. Overall, this study showed that excessive overtime work was most associated with Extraversion among Big Five factors. We propose that Extraversion should be taken into consideration preferentially to control overtime work. For example, if we devise practical ways to enhance communication among workers or train them in communication skills it may induce sharing of heavy workloads resulting in reduction of excessive overtime work in individuals during busy periods. Moreover, this intervention may contribute to improvement of workers’ mental health.

Although personality can be expected to affect work time, there have been few reports regarding this association. However, as number of work hours is known to be a key factor in adverse health effects, it is necessary to discuss these associations. The lack of reports is probably due to the absence of accurate work time data because previous studies used self-reported number of work hours and average number of hours per day [6,27]. Therefore, accurate data regarding overtime work hours throughout the year could not be considered. This important issue was noted as a problem that should be clarified in a previous report [14]. In the present study, we used precise work hour data and evaluated the association with personality because we used work time records. Moreover, this study suggested that excessive overtime is a useful factor for work time analysis that can also be measured as accumulation of number of working hours over short periods. Therefore, this outcome measure allowed the evaluation of more information than just average number of work hours. Thus, it was suggested that not only average number of work hours but also occurrence of excessive overtime work should be taken into consideration to evaluate work time effects.

This study had several limitations. First, because this pilot study was cross-sectional, cause-effect relations were less clear than had it been longitudinal. In addition, time-course change of the relations between variables and number of working hours was also unclear. Because time-course change of the variables may affect the association between personality and number of working hours, a longitudinal study will be necessary. Second, the alpha of the work-time related questionnaire was not high although the questions were based on those validated in previous studies. This may be because the questionnaire included some questions which were not entirely associated with number of working hours. Improvement of the questionnaire is a task for the future. Third, to the extent that the sample size was relatively small and drawn from white-collar workers at one institution it may have influenced the skewness or distribution of the personality scores. Indeed, some employers screen and select employees that fit a prescribed or desired personality profile deemed most suitable for their organization, which would explain any skew in distribution of personality scores. In addition a larger sample from a wider occupation population might have allowed us to evaluate extremely excessive overtime work such as over 80 h/month or 100 h/month. Further studies in larger numbers of subjects across a wider range of occupations are necessary to fully generalize the results. Moreover, selection bias may have occurred because those that worked longer work hours may have been suspicious and chosen not to participate. Response rates should be increased in future studies to avoid such bias. Fourth, the possibility of underestimation of number of working hours could not be fully ruled out. In Japan, work hours data from Labour Force Survey [18] is usually slightly larger than that from Monthly Labour Survey [19]. This disparity may be due to unpaid overtime work because the latter was derived from company data. More precise methods to investigate number of working hours should be determined. As issues remain unclear in this field, further studies in several disciplines are necessary.

## Conclusion

The results of the present pilot study indicate low Extraversion is associated with excessive overtime work. We suggest personality factors should be included in studies which evaluate number of working hours and mental health. Moreover, strengthening communication skills or training workers in skills comprising Extraversion factors may contribute to reduction of excessive overtime work in individuals resulting in positive health benefits. Further studies are needed to clarify associations among personality, long work hours, and mental health among workers.

## Competing interests

The authors declare that they have no competing interests.

## Authors’ contributions

MU contributed the study design, data collection, analysis, interpretation, and manuscript preparation. MK and SK helped with manuscript preparation and interpretation. All authors read and approved the final version of the manuscript.
